# Supplementary material for: Optogenetic inhibition of Delta reveals digital Notch signalling output during tissue differentiation
Source: EMBO Rep. 2019 Oct 31;20(12):e47999. doi: 10.15252/embr.201947999 (PMC6893285; doi:10.15252/embr.201947999)
Supplement: Supplementary file 1 — Expanded View Figures PDF [file EMBR-20-e47999-s001.pdf]

Expanded View Figures

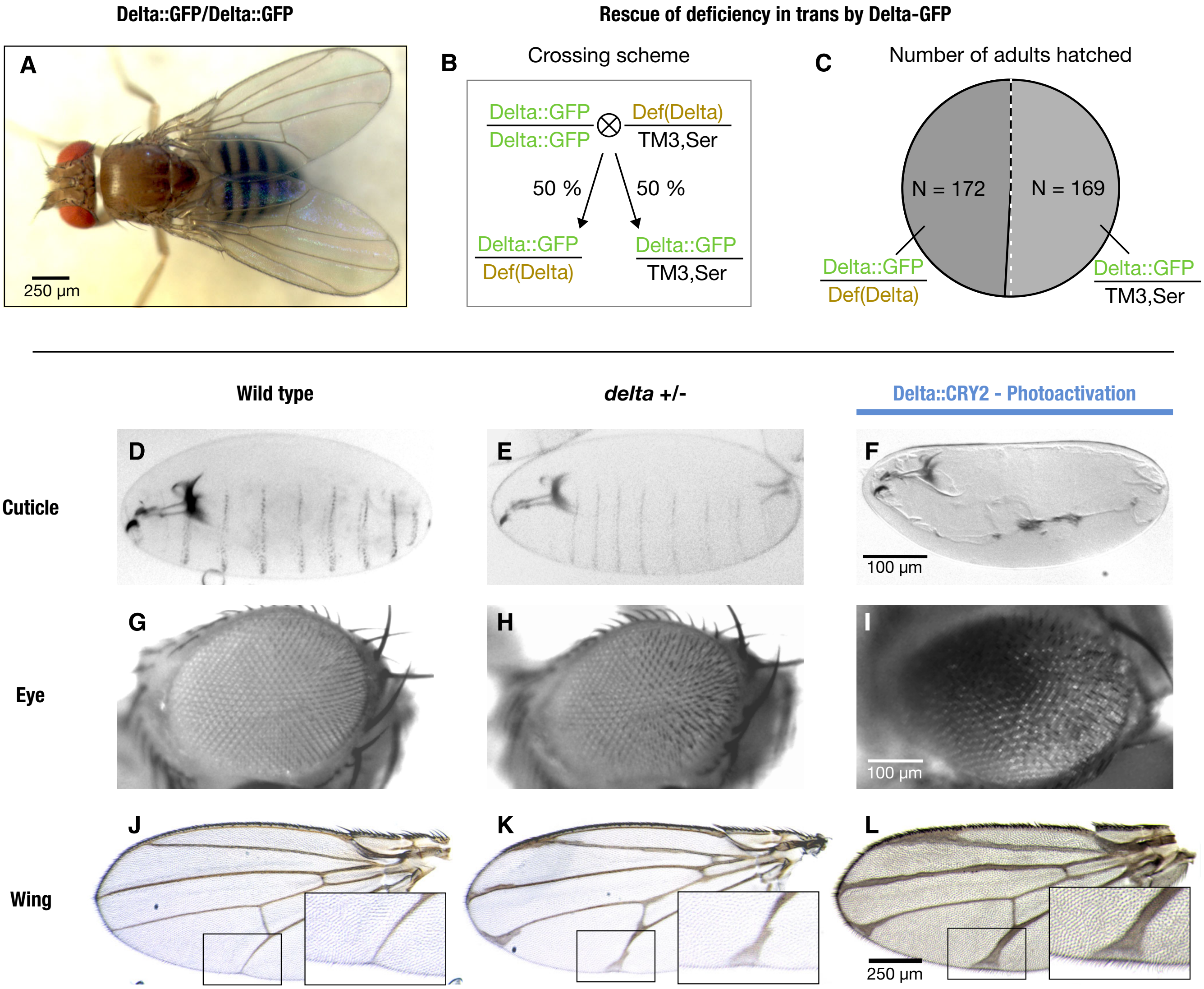

Figure EV1. Characterization of Delta::GFP and Delta::CRY2 homozygous flies.

A–C Delta::GFP flies are homozygous viable (A), and the Delta GFP allele was able to rescue a Delta deficiency *in trans* (B, C). When Delta::GFP homozygous flies were crossed to flies heterozygous for a Delta deficiency, the genotype frequency of adults that hatched in the F1 generation (Delta::GFP/Def(Delta) : Delta::GFP/TM3, Ser) closely matched the expected ratio of 50:50. Total number of adults hatched, *N* = 341 (*N* = 172 for Delta::GFP/Def(Delta) and *N* = 169 for Delta::GFP/TM3, Ser).

D–L Benchmarking of light-induced loss-of-function phenotypes in Delta::CRY2 flies. Delta::CRY2 flies reared in the light exhibit more severe Delta loss-of-function phenotypes compared to Delta heterozygotes. This is evident from the loss of denticle belt patterning in the embryonic cuticle (F), disorganization of the ommatidia (I) and thickening of the wing veins (L) when compared to the Delta heterozygous counterparts where the denticle belt patterning is unaffected (E), and the ommatidia (H) and wing venation (K) phenotypes are milder. Note, panels (F, I, L) are the same as in Fig 1. Corresponding wild-type cuticle, eye and wing are depicted in panels (D), (G) and (J), respectively. Scale bars, 100 µm in (F) and (I), and 250 µm in (L).

© 2019 European Molecular Biology Laboratory

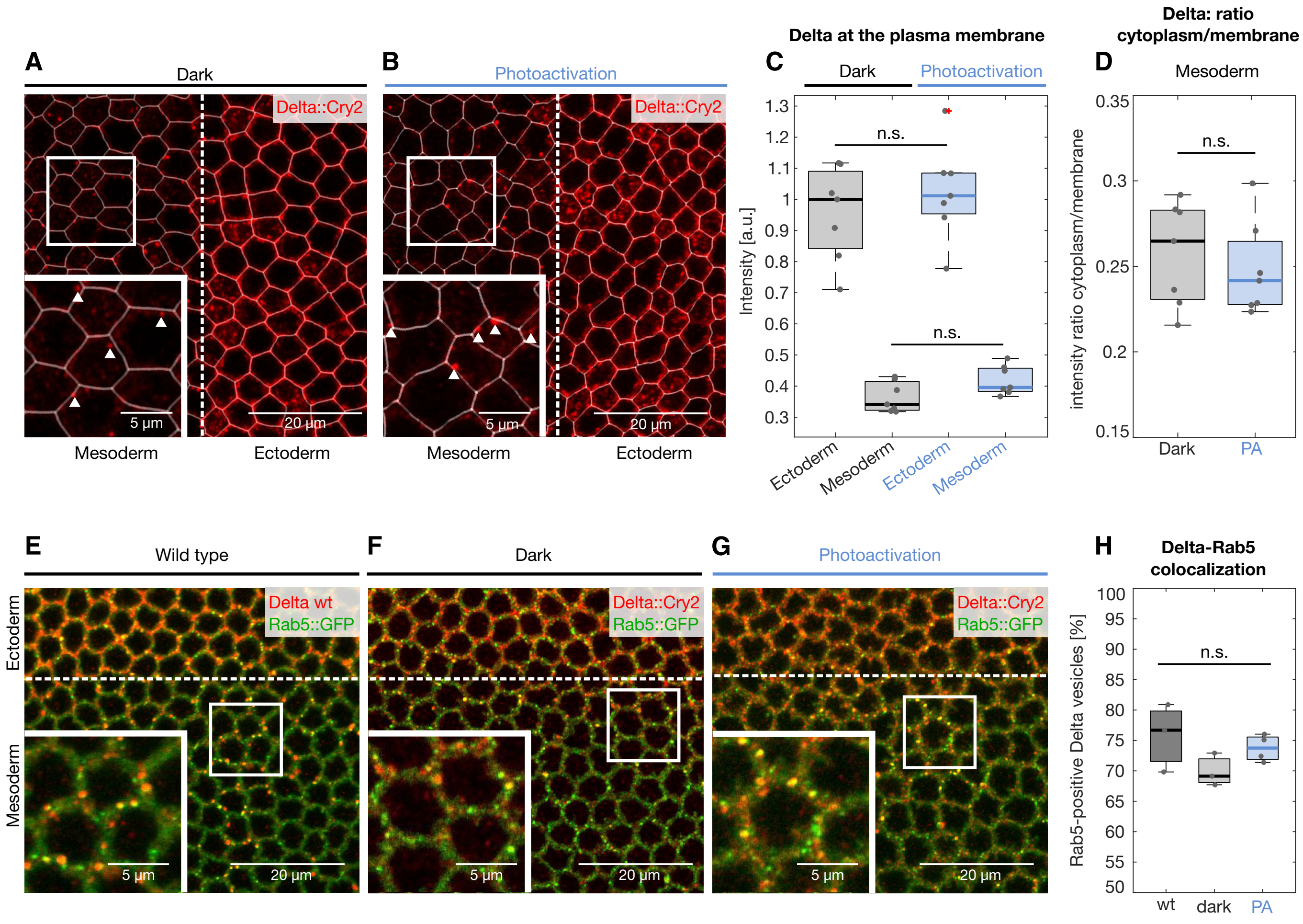

Figure EV2. Light-induced Delta clustering does not affect Delta trafficking in the mesoderm.

A–C Plasma membrane segmentation of Delta::CRY2 embryos immunostained with anti-Delta (red) and anti-Cadherin (used for plasma membrane segmentation, white) antibodies at the end of cellularization, in embryos fixed in the dark (A) or after batch photo-activation (B). Panels display single confocal *z*-slices. Magnified insets in (A, B) show Delta cytoplasmic vesicles (indicated with white triangles) in the mesoderm that are excluded from the plasma membrane segmentation. Plot (C) shows the quantification of Delta plasma membrane levels in the ectoderm and mesoderm in both dark and photo-activated conditions. In both cases, Delta plasma membrane levels were depleted by ~70% in the mesoderm compared to the ectoderm. No significant difference was observed when comparing ectodermal or mesodermal Delta plasma membrane levels with or without photo-activation, indicating that Delta was normally depleted in both conditions. $N = 7$ embryos for both dark and photo-activation, n.s. represents no statistically significant difference in a 2-sample *t*-test. In the box plots, the solid horizontal line, bottom and the top edge of each box indicate the median, the 25th percentile and 75th percentile, respectively. Whiskers extend to the most extreme data point. “+” symbols indicate outliers. Scale bars 20 μm and in insets 5 μm (A, B).

D Plot depicting the cytoplasm to membrane ratio of Delta in the mesoderm, both in the dark and upon photo-activation. No significant difference was observed between both conditions. $N = 7$ embryos for both dark and photo-activation, $n = $ ~40 cells/embryo. n.s. represents no statistically significant difference in a 2-sample *t*-test. In the box plots, the solid horizontal line, bottom and the top edge of each box indicate the median, the 25th percentile and 75th percentile, respectively. Whiskers extend to the most extreme data point.

E–H The extent of colocalization of Delta-positive vesicles with the early endosomal marker Rab5 did not change upon light-induced Delta clustering. Single confocal z-slices of Rab5::GFP (E), Rab5::GFP in a Delta::CRY2 homozygous background embryos either fixed in the dark (F) or after batch photo-activation (G). In all panels, Delta was visualized by immunostaining using an anti-Delta antibody (red). The endogenous GFP signal was used to visualize Rab5 (green). Magnified insets in all three panels show colocalization between Delta-positive vesicles and Rab5 (E–G). Plot (H) shows the extent of colocalization of Delta- and Rab5-positive vesicles. ~75% of Delta-positive vesicles colocalized with Rab5, and this value did not significantly differ from wild-type, Delta::CRY2 (photo-activated) or Delta::CRY2 (dark) conditions. $N = 4$ embryos for photo-activation and $N = 3$ embryos for wild-type and dark conditions. n.s. represents no statistically significant difference in a 2-sample *t*-test. In the box plots, the solid horizontal line, bottom and the top edge of each box indicate the median, the 25th percentile and 75th percentile, respectively. Whiskers extend to the most extreme data point. Scale bars, 20 μm and in insets 5 μm (E–G).

© 2019 European Molecular Biology Laboratory

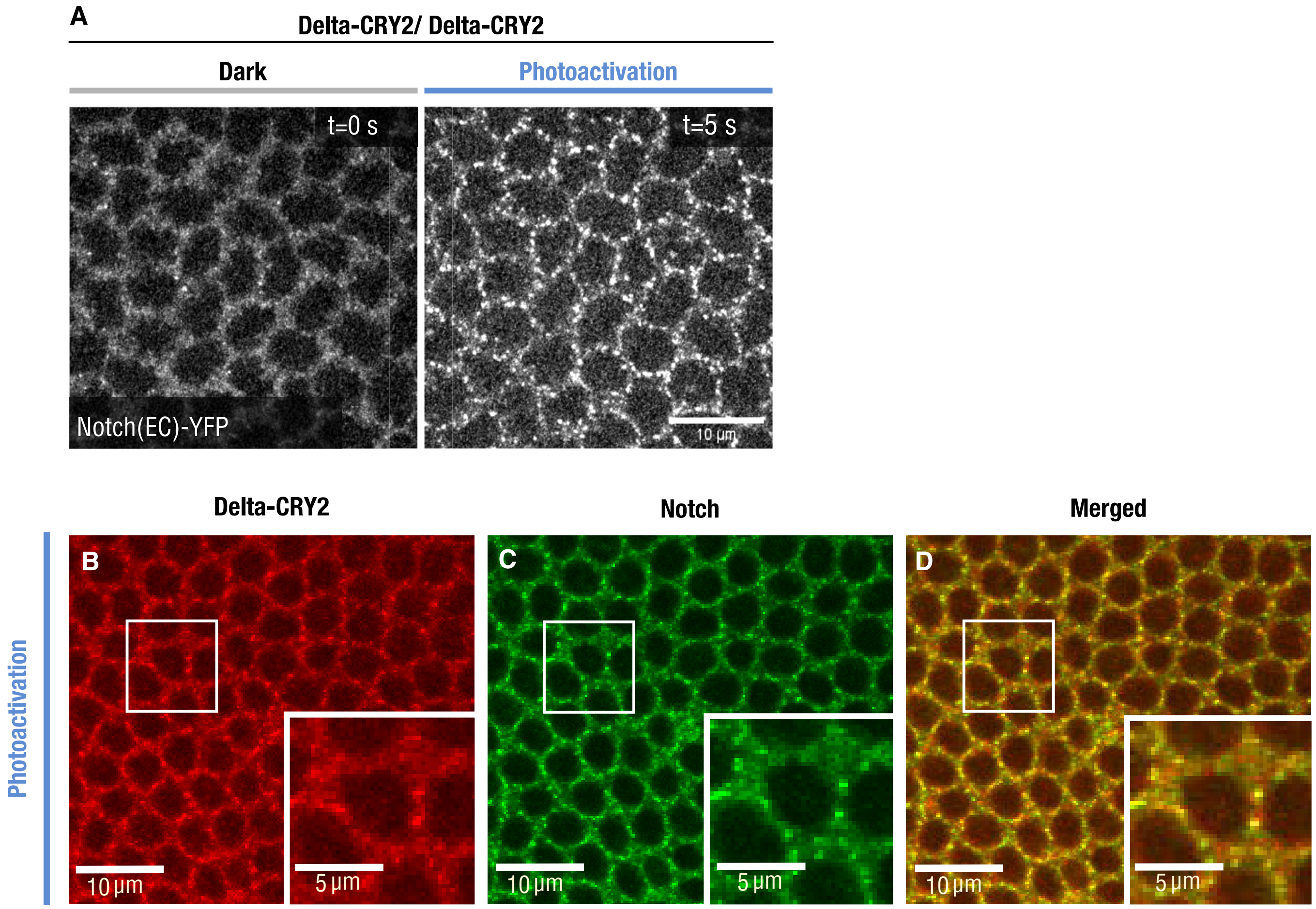

Figure EV3. Notch clustering in a Delta::CRY2 homozygous background.

A Single confocal *z*-slices of cellularizing embryos expressing endogenously tagged Notch::YFP imaged using an argon laser (λ = 514 nm) in a Delta::CRY2 homozygous background before and after photo-activation with a stack of size *z* = 10 µm for a duration of 5 s (λ = 488 nm, 0.6 mW). Note that Notch clustering in Delta::CRY2 homozygous background is faster than in Delta::CRY2 heterozygous see Fig 2H. This is probably due to the higher density of the Delta::CRY2 molecules. Scale bar, 10 µm.

B–D Delta::CRY2 clusters in the ectoderm contain Notch. Immunostaining of embryos expressing Notch::YFP in a Delta::CRY2 homozygous background using anti-GFP (targeting Notch::YFP) and anti-Delta. Delta (B), Notch (C), overlay (D) after batch photo-activation (λ = 488 nm), as described in the methods. Panels display max. intensity *z*-projections of three slices at a *z*-interval of 0.7 µm. Magnified insets show ectodermal clusters of Delta::CRY2 at the plasma membrane (B) co-clustering of Notch (C), and the merged image in (D). Scale bar, 10 µm and in insets 5 µm.

 © 2019 European Molecular Biology Laboratory

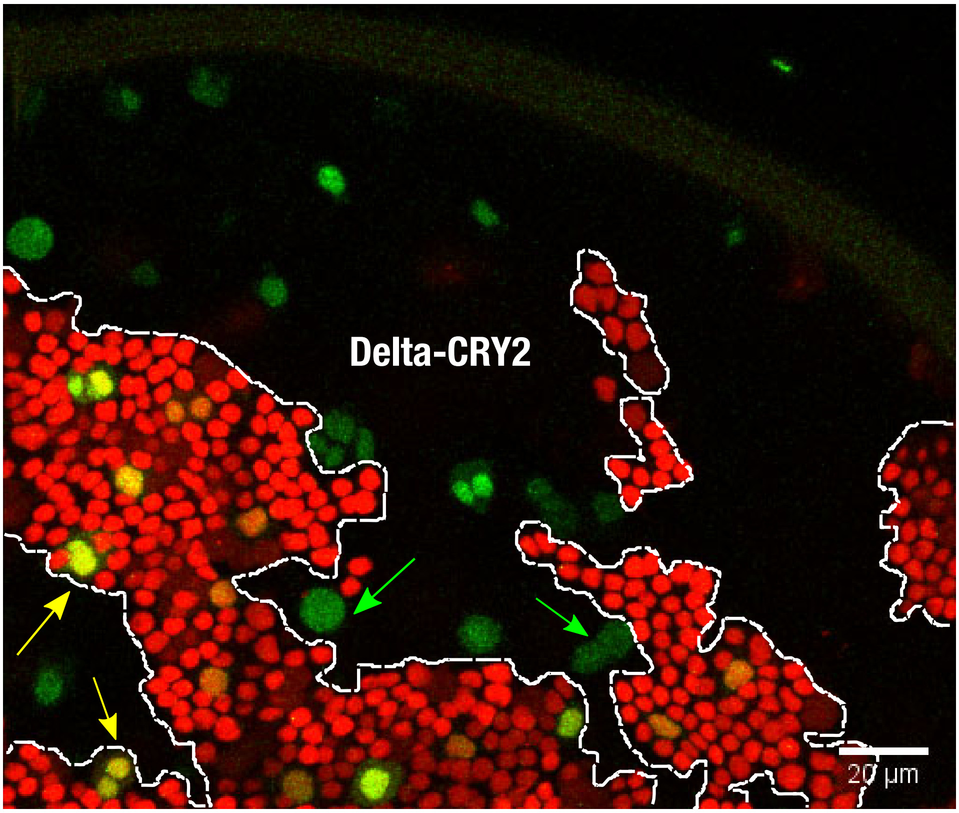

Figure EV4. Analysis of sensory organ precursor (SOP) fate decisions across Delta::CRY2/wild-type clone borders.

Control (no photo-activation) notum at 18 h after puparium formation. Maximum-intensity *z*-projections of 4 slices at 1-µm *z*-interval showing Delta::CRY2 clones (lack of nls-RFP), and dashed lines depicting the clone borders where the number of SOPs (green) are scored. As described in Fig 3O, in the dark, ~60% of SOPs are present inside the Delta::CRY2 clone. Yellow arrows indicate examples of SOPs inside the wild-type clone, and green arrows show SOPs scored inside the Delta::CRY2 clone. Scale bar, 20 µm.

© 2019 European Molecular Biology Laboratory

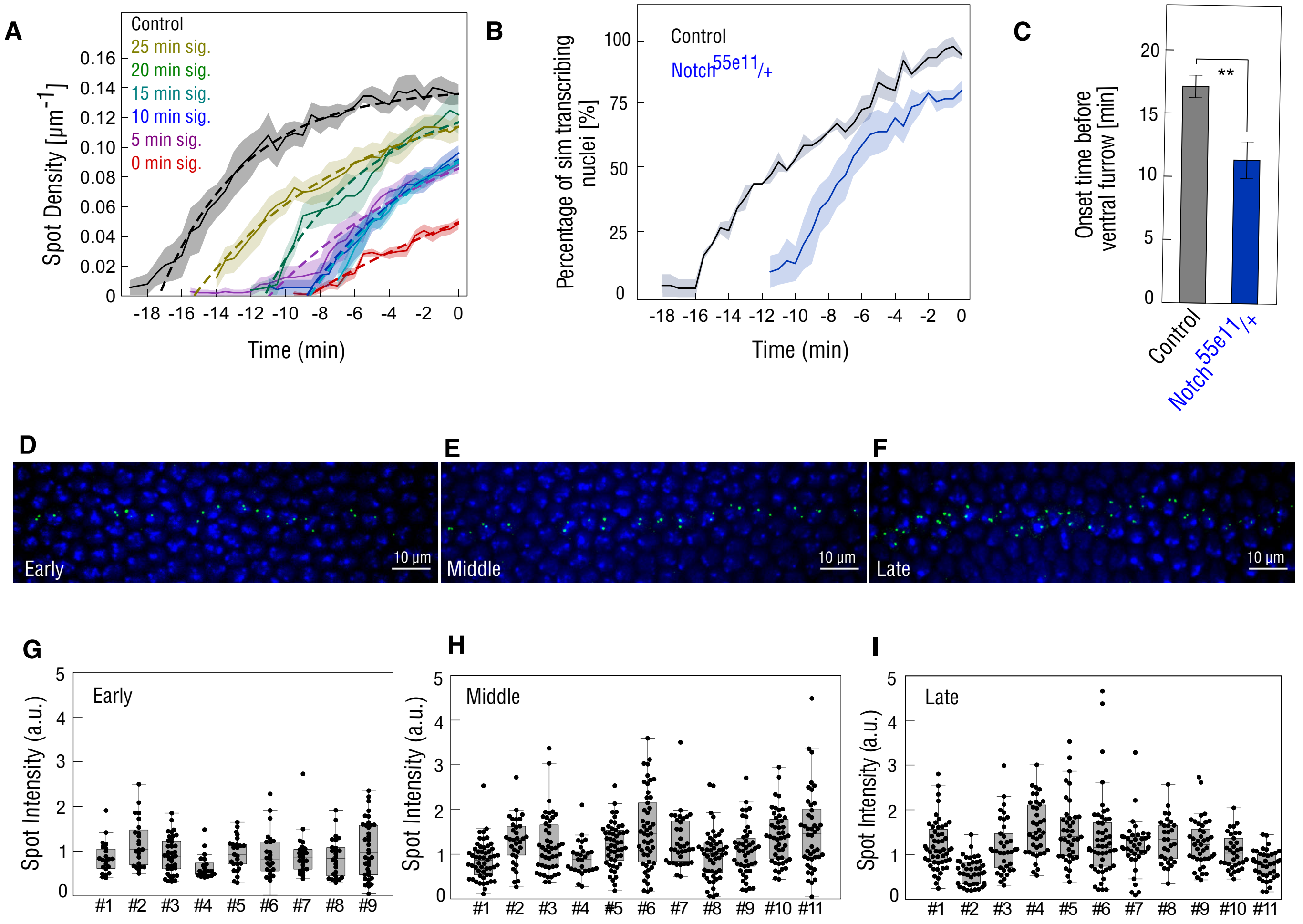

Figure EV5. Quantification of *sim* transcription during mesectoderm specification.

A Fitting of *sim* spot-density curves for estimation of activation rates and delays.
The average spot-density curves (number of sim spots/unit length of embryo, μm^{-1}) for each condition (control/25 min/20 min/15 min/10 min/5 min/0 min signalling) corresponding to Fig 4E were adjusted by N_{max} $(1-\exp(-k\ (t-t_0))$, where N_{max} is the maximum number of spots that can be activated (i.e. the number of nuclei in the mesectoderm), k is rate at which mesectodermal nuclei start to transcribe *sim* and t_0, the delay in the onset of spot appearance with respect to the control. N_{max} was taken as constant, and only two parameters k and t_0 were adjusted for each curve. Solid lines represent the mean, dashed lines represent the fitting of the mean curve, and shaded areas represent the standard error of mean.

B, C Reducing the copy number of Notch results in a delay of *sim* transcription. Plot depicting the percentage of *sim* transcribing nuclei over time in embryos carrying one mutant allele of Notch, Notch^{55e11} compared to controls (B). Solid lines represent the mean, and shaded areas represent the standard error of mean. Bar plot showing that onset of *sim* spot detection in Notch^{55e11} heterozygous embryos is significantly delayed compared to controls (C). The onset of ventral furrow formation is considered as t = 0 min. N = 3 embryos for both conditions. $^{**}P \leq 0.01$, two-sample t-test. Error bars represent standard error of mean.

D–I Fluorescence *in situ* hybridization of *sim* transcription. Representative maximum-intensity *z*-projections depicting nascent *sim* transcripts (green) in the mesectoderm in wild-type (w1118) embryos staged as early (D), middle (E) and late (F) during cellularization, as described in the methods section. Nuclei were stained with DAPI (blue). Scale bar, 10 µm. (G–I) Box plots showing the quantification of nascent *sim* transcripts across wild-type embryos during early (G), middle (H) and late cellularization (I). There was only a 25% variation in sim spot intensity between time-points, a value that was smaller than the ~40% variation in sim spot intensity within single embryos. In the box plots, the horizontal line, bottom and the top edge of each box indicate the median, the 25th percentile and 75th percentile, respectively. Whiskers extend to 1.5 times the interquartile range. N = 9 embryos for the early stage and N = 11 embryos for both the middle and late stages.

© 2019 European Molecular Biology Laboratory
