# Supplementary material for: Optogenetic inhibition of Delta reveals digital Notch signalling output during tissue differentiation
Source: EMBO Rep. 2019 Oct 31;20(12):e47999. doi: 10.15252/embr.201947999 (PMC6893285; doi:10.15252/embr.201947999)
Supplement: Supplementary file 2 — Movie EV1 [file EMBR-20-e47999-s002.zip › Movie_EV1/movie_EV1.docx]

**Movie EV1. Light-induced Delta::CRY2clustering.**

Confocal movie of a Delta::GFP::CRY2 heterozygous embryo (ectoderm stage 5) corresponding to figure 2 (B). Simultaneous photo-activation and imaging was done at a time resolution of 2 s with an argon laser (λ= 488 nm, 0.6 mW). Scale bar, 10 µm.
