# Supplementary material for: Optogenetic inhibition of Delta reveals digital Notch signalling output during tissue differentiation
Source: EMBO Rep. 2019 Oct 31;20(12):e47999. doi: 10.15252/embr.201947999 (PMC6893285; doi:10.15252/embr.201947999)
Supplement: Supplementary file 3 — Movie EV2 [file EMBR-20-e47999-s003.zip › Movie_EV2/movie_EV2.pdf]

Movie EV2. *sim*-MS2 expression in a control embryo. Confocal movie shown as maximum intensity projections of 63 slices at 0.4 µm z-interval of a control embryo (non Delta::CRY2) which was photo-activated for 60 min from the onset of cycle 14. Image acquisition (λ= 488 nm) was started when *sim* spots were visible and was continued until the onset of ventral furrow formation at a time-resolution of 30 sec. Scale bar, 10 µm.
