# Supplementary material for: Optogenetic inhibition of Delta reveals digital Notch signalling output during tissue differentiation
Source: EMBO Rep. 2019 Oct 31;20(12):e47999. doi: 10.15252/embr.201947999 (PMC6893285; doi:10.15252/embr.201947999)
Supplement: Supplementary file 5 — Movie EV4 [file EMBR-20-e47999-s005.zip › Movie_EV4/movie_EV4.docx]

**Movie EV4: *sim*-MS2 expression in a Delta::CRY2 embryo, 20 min signaling.**

Confocal movie shown as a maximum intensity projections of 63 slices at 0.4 µm z-interval of a Delta::CRY2 embryo in which signaling was allowed for 20 min according to the photo-activation scheme in Figure 4(D) and described in the methods. Image acquisition (λ= 488 nm) was started when *sim* spots were visible and was continued until the onset of ventral furrow formation at a time-resolution of 30 sec. Scale bar, 10 µm.
